# Reversible pH Stimulus-Response Material Based on Amphiphilic Block Polymer Self-Assembly and Its Electrochemical Application

**DOI:** 10.3390/ma9060478

**Published:** 2016-06-15

**Authors:** Tianyi Wang, Hongmei Zhu, Huaiguo Xue

**Affiliations:** College of Chemistry and Chemical Engineering, Yangzhou University, 180 Si-Wang-Ting Road Yangzhou, Yangzhou 225009, China; yzwangtianyi@foxmail.com (T.W.); yzdxzhm@sina.cn (H.Z.)

**Keywords:** microporous thin film, self-assembly, PS–*b*–PAA, block polymers, stimulus-responsive, surface analysis

## Abstract

Stimulus-responsive microporous solid thin films were successfully fabricated by simple molecular self-assembly via an amphiphilic block polymer, polystryene–*b*–polyacrylic acid (PS–*b*–PAA). The solid thin films exhibit different surface morphologies in response to external stimuli, such as environments with different pH values in aqueous solutions. The experiments have successfully applied atomic force microscope (AFM) technology to observe *in-situ* surface morphological changes. There is a reversible evolution of the microstructures in buffer solutions over a pH range of 2.4–9.2. These observations have been explained by positing that there is no conventional PAA swelling but that the PAA chains in the micropores stretch and contract with changes in the pH of the solution environment. The hydrophobicity of the solid thin film surface was transformed into super-hydrophilicity, as captured by optical contact angle measurements. The stimulus-responsive dynamics of pore sizes was described by a two-stage mechanism. A promising electrochemical application of this film is suggested via combination with an electrochemical impedance technique. This study is aimed at strategies for the functionalization of stimulus-responsive microporous solid thin films with reversible tunable surface morphologies, and exploring new smart materials with switch-on/switch-off behavior.

## 1. Introduction

Stimulus-responsive microstructures are new functional materials for important devices, which have received significant attention because of a wealth of potential applications, such as sensors, membranes, micro-actuators, microfluidic devices, drug delivery systems, and other advanced microdevices [1,2,3]. Environmentally sensitive polymers have received a great deal of attention in recent years, and their synthesis and applications have become a major research field in polymer science [4,5,6]. Polymeric materials, in particular, can respond to environmentally sensitive stimuli (such as the solvent, temperature, pH, light, ionic strength, *etc.*) [7,8,9] by cooperative conformational changes of the microstructures [10]. The field of stimulus-responsive materials, and of polymers in particular, is relatively new, and it has relevance to the field of nano-science or nano-engineering [11,12,13,14]. Smart responsive polymer films can spontaneously alter or change their surface properties, such as wettability, reactivity, or biocompatibility. Some recent developments have been aimed at strategies for the functionalization of surfaces with stimulus-responsive polymer brushes [15,16]. Some microstructures prepared from weak polyelectrolytes provide versatile surfaces that can respond to changes in temperature, solvent, pH, and other stimuli, which suggest that weak polyelectrolytes are potential “active” components in the microstructure of smart surfaces [17,18,19,20,21,22,23,24]. In the fabrication of stimulus-responsive microporous thin films, poly(acrylic acid) (PAA) is an excellent candidate with changes in the external pH and with the different degrees of ionization of the carboxyl groups [25,26,27,28,29].

Microporous polymer membranes (pore size 0.01–20 µm) are widely used in industry, medicine, pharmacology, and in research on the separation and concentration of particles, colloids, proteins, enzymes, and cells [30,31]. Since Francois *et al.* as pioneers fabricated microporous solid thin films by simple molecular self-assembly [32], the preparation of elaborate functional honeycomb-structured polymer films based on “breath figure” has attracted many researchers [33,34,35,36], and the applications of hierarchical and functional honeycomb films in separation, biocatalysis, biosensing, templating, stimuli-responsive surfaces, and adhesive surfaces have been investigated and summarized [37,38,39].

Monitoring and recording the *in-situ* transformation of stimulus responsive are still challenges. Matyjaszewski *et al.* utilized an atomic force microscopy (AFM) to investigate conformations of a series of water-soluble loosely grafted PAA brushes under different pH conditions [40]. Escale *et al.* employed AFM and optical contact angle (OCA) to demonstrate the hierarchical structures of smart porous polymer films based on a pH-responsive polystyrene-block-poly(4-vinylpyridine) [41]. Minko *et al.* reported pH dependent swelling of cross-linked poly(2-vinylpyridine) membranes and pH influences on the micropore size [18]. However, their work was not based on *in-situ* AFM images.

Previously, we have reported facile fabrications of a microporous solid thin film based on PS–*b*–PAA self-assembly, an investigation of its tunable surface properties, and their electrochemical applications [42,43,44,45]. In this paper, we demonstrate surface morphology changes of the microporous solid thin film in response to external pH stimuli by *in-situ* AFM. Reversible evolution of the microstructure in aqueous solutions (pH range of 2.4–9.2) was observed. The stimulus-responsive dynamics of pore size changes was discussed. A promising electrochemical application based on an electrochemical impedance technique was presented. Our study is aimed at strategies for the functionalization of stimulus-responsive smart materials with tunable surface morphologies and their applications.

## 2. Experimental Section

### 2.1. Materials

**Scheme 1:**
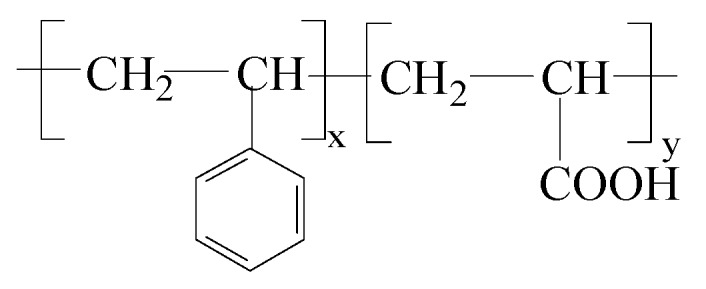
Chemical structure of PS–*b*–PAA.

The PS–*b*–PAA (Scheme 1) was obtained from Zhejiang University, China. The blocks of the copolymer consisted of 90 (x) and 45 (y) monomers for the hydrophobic PS block and the hydrophilic PAA block, respectively. The molecular weight was found to be 12,500 g/mol with block weights, PS (9270)-*b*–PAA (3230). The dispersity (D) with a symmetrical molar mass distribution is 1.05. Tetrahydrofuran (THF) was obtained from the Chemical Reagent Company of Shanghai (Shanghai, China). They were used as received without further purification. All the other reagents used were analytical grade. Deionized water was obtained by purification through a Millipore water system and was used throughout. All the experiments were conducted at room temperature.

A glass substrate (1 × 1 cm^2^) was immersed in a piranha solution (98% H_2_SO_4_/30% H_2_O_2_, volume ratio, 3:1) at 80 °C for 2 min, and then rinsed with deionized water and ethanol before film fabrication. (Caution: Piranha solution reacts violently with many organic materials and should be handled with care). Finally, it was dried with a stream of high purity nitrogen. In a humid atmosphere (the relative humidity, which is defined as the percentage obtained by dividing the vapor pressure of water in this system by the saturated vapor pressure of pure water, is above 50% and maintains constant during the film preparation), 50 μL of a PS–*b*–PAA/tetrahydrofuran (THF) solution (10 mg/mL) was cast onto the surface of the glass with a micro-syringe at the temperature of 25 °C.

### 2.2. Apparatus

SEM images of PS–*b*–PAA microporous film were obtained with a XL-30E scanning electron microscope (Philips, Eindhoven, The Netherlands). The electron gun voltage was set at 20 kV, and the sample surface was sputter coated with gold to achieve a better quality of the secondary electron images under the electron beam.

AFM images of the films were obtained by a Multi–Mode Nanoscope IIIa scanning probe microscope (Veeco Company, Plainview, NY, USA) in contact mode using a micro-fabricated silicon nitride probe (spring constant *k* = 0.58 N/m). A feedback loop maintains a constant deflection between the cantilever and the sample to conduct a “set-point” deflection. The data of pore sizes referred to the apparent diameters observed from the top view of the film. The pore depth data was obtained from SEM of the cross-section of the microporous film. The surface coverage, pore-pore distance, and apparent diameter of the as-prepared film were all calculated by Image-pro Plus 6.0 (Media Cybernetics Company, Silver Spring, New York, NY, USA), and sampling population and distribution of the reported pore values was also obtained from large scale morphology images.

For AFM imaging in aqueous environments, solid thin films of PS–*b*–PAA on the glass substrate were investigated in a fluid cell. pH–responsive PS–*b*–PAA microporous films of an area were investigated *in-situ* in the fluid cell containing the buffered solution. Prior to each measurement, the films were equilibrated for 40 min at a given pH value. To maintain constant counter-ion type and ionic strength, sodium phosphate buffer solutions (H_3_PO_4_/NaH_2_PO_4_/Na_2_HPO_4_/Na_3_PO_4_) were prepared with pH values ranging from 2.4 to 9.2 at fixed buffer strength (0.05 M). Before our experiments, the fluid cell and O-ring were carefully cleaned. To investigate structural evolution as a function of different pH solutions, films were immersed in a liquid cell containing different pH solutions, and *in-situ* scans were performed after 40 min as an interval in the same area. The volume of the fluid cell was so small (about 50 µL) that an existing liquid can be completely replaced by a new one soon after injection of the different liquid into the fluid cell. The wettability of the PS–*b*–PAA microporous solid thin films was characterized by an Optical Contact Angle 40 instrument (Dataphysics, Filderstadt, Germany), using the sessile water droplet method. A water droplet size of 4 μL was used for optical contact angle measurements. Video system: CCD video camera with a resolution of 768 × 576 pixels and up to 50 images per second. All measurements described above were performed at room temperature.

A glassy carbon electrode (GCE, diameter of 3.0 mm) was polished before each casting experiment with 1, 0.3, and 0.05 µm α-alumina powders, respectively, rinsed thoroughly with deionized water between each polishing step, sonicated in 1:1 nitric acid, acetone, and deionized water, successively. Finally, it was dried with a stream of high-purity nitrogen immediately before use. PS–*b*–PAA was dissolved in THF at a typical concentration of 10 mg/mL. One drop of the PS–*b*–PAA/THF solution was dropped onto the polished GCE surface. Then, the as-prepared GCE was placed in a closed system. The humidity resulted from water vapor through the air flow. A hygrometer was settled in the closed system in order to investigate the variation of the humidity. Then, the GCE was rotated at 2000 rpm for the spin-coating process, which lasted for 10 min. The atmosphere in the closed system was kept with constant humidity, which was about 60%. The electrochemical impedance spectra (EIS) were performed on an Autolab electrochemical analyzer (Eco Chemie, Utrecht, The Netherlands) equipped with a one-compartment three-electrode system which consists of a GCE modified by a PS–*b*–PAA thin microporous film as the working electrode, a saturated calomel electrode (SCE) as the reference electrode, and a platinum electrode as the counter electrode. EIS experiments were measured in the frequency range of 10^−1^ to 10^6^ Hz, with an open circuit potential, a single modulated AC potential of 10 mV, and in a solution of 5.0 mM hexacyanoferrate (II)/(III) containing 0.1 M KCl.

## 3. Results and Discussion

### 3.1. Fabrication of Microporous Solid Thin Films

**Figure 1:**
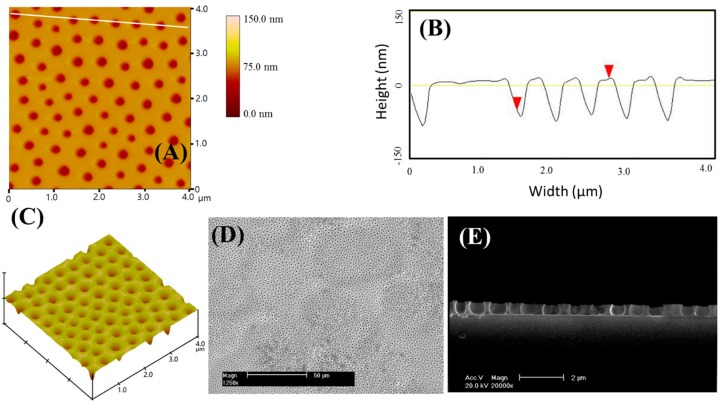
(**A**) AFM image of PS–*b*–PAA microporous solid thin film; (**B**) Cross-section of the PS–*b*–PAA microporous solid thin film; (**C**) Three-dimensional AFM image of PS–*b*–PAA microporous solid thin film; (**D**) SEM image of PS–*b*–PAA microporous solid thin film; (**E**) SEM image of the cross section of the PS–*b*–PAA microporous solid thin film. The concentration of the PS–*b*–PAA/THF solution: 10 mg/mL; the relative humidity: 60%.

The surface morphology of the three-dimensional microporous solid thin film formed on the glass slide is shown in Figure 1. The AFM image of the film is shown in Figure 1A, which shows the microporous morphology of the film. In addition, the pore surface coverage is about 20%. The average pore to pore distance of the film was measured to be 0.72 μm with RSD 11%. According to the AFM image (Figure 1A) and the section curve (Figure 1B), the pore size of the PS–*b*–PAA porous polymer films amounts to about 0.24 ± 0.08 μm (*n* = 11, confidence level 95%) according to top view analysis. For more detailed insight into the geometry, we display an AFM image of a three-dimensional structure in Figure 1C. A typical SEM image of the PS–*b*–PAA microporous solid thin film is presented in Figure 1D. As shown in Figure 1E, the investigation of the depth of the micropore is presented by a SEM image of the section of the film, in which the pore depth can be observed with 0.7 ± 0.1 μm.

### 3.2. pH Responses of PS–b–PAA Microporous Films with Their Tunable Surface Properties

We examine the influence of the pH value of the aqueous solution on the morphology and the pore sizes of the film by *in-situ* AFM. The equilibrium time of the pores at different pH values was tested, which was shown in Table 1, and the pores at all pH values can achieve equilibrium within 60 min. Therefore, to ensure equilibrium morphologies, images were obtained after immersion for 60 min. The AFM topographical images reveal an increase in the pore size that occurred with a decrease in acidity of the aqueous solution.

**Figure 2:**
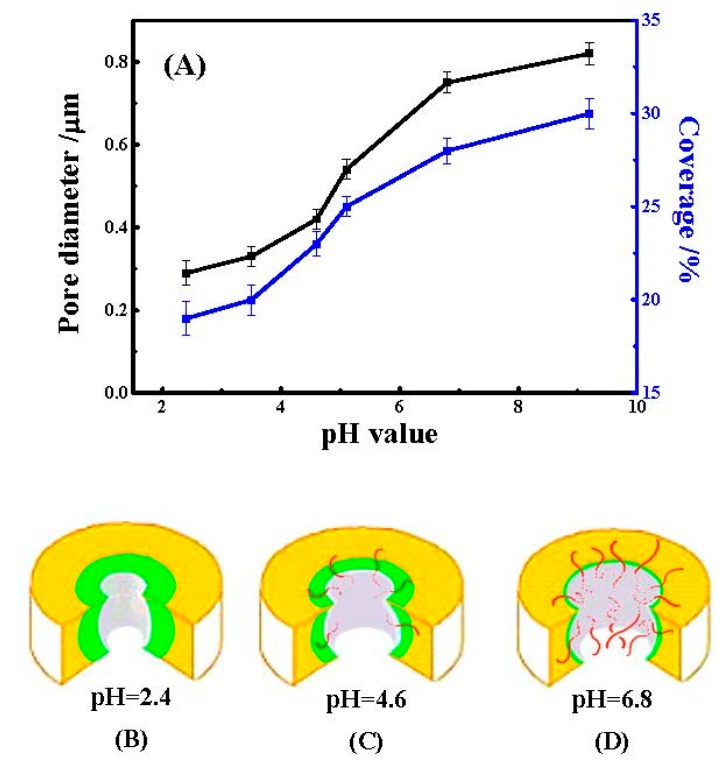
(**A**) The variation trend of pore size (black line) and surface coverage (blue line) according to Figure 1D, and the y-axis on the left refers to the pore diameter (μm), while the y-axis on the right indicates the percentage of pore coverage (%); (**B**–**D**) are schematic drawings of the PS–*b*–PAA microstructures in pH < 4.0, 4.0 < pH < 6.0, and pH > 6.0, respectively. The phases were assigned to the PS matrix (orange domain), the PAA domain (green domain), and the stretched PAA chains (red dotted lines).

It was revealed that an increase in the pore size occurred with a decrease in acidity in the aqueous solution. Qualitatively, a top view analysis of the AFM topographical images indicates that the pore sizes enlarged accordingly with the increasing pH values. The variation tendency of pore sizes and surface coverages showed a similar tendency, as can be seen in Figure 2A.

It is well-known that asymmetric, amphiphilic block copolymers can self-assemble to form aggregates in selective solvents [46]. The PS–*b*–PAA amphiphilic block copolymer can form micelles with a hydrophilic core (PAA) and a hydrophobic corona (PS) in low polar media such as THF, while aggregates with the opposite composition are present in solvents with high polarity such as water. After the solvent evaporates, there is a process of phase inversion where water droplets condense onto the solution surface. Therefore, each micropore is enclosed by a PAA domain, which has many carboxylic functional groups (–COOH). Every annular PAA domain is surrounded by a PS matrix [18]. Schematic diagrams of the micropores were constructed in Figure 2B–D. Because the polymer PAA is a weak polyelectrolyte with a p*K*_a_ of 4.6 [47], the degree of dissociation of the carboxylic acid groups depends on the pH value of the aqueous solution. For pH < p*K*_a_, PAA chains are slightly stretched and dispersed by absorbing water in aqueous solutions because of the inability of the carboxylic acid groups to ionize, since carboxylic acid groups exist as –COOH. However, for pH > p*K*_a_, PAA chains are dramatically stretched and dispersed because most carboxylic acid groups are dissociated, and electrostatic repulsive forces among ionized carboxylate anions are increased. The degree of stretching of the PAA chains depends on the degree of ionization of the PAA or the pH value of the medium. In our experiment, the pH response of PS–*b*–PAA microporous solid thin films was studied by carrying out *in-situ* AFM in a liquid cell containing a 0.05 M sodium phosphate buffer solution.

At low pH (pH < 4.0), PAA chains are partially stretched and dispersed by absorbing water in aqueous solutions, while the compact skeleton structure is composed of un-dispersed PAA chains and PS matrix (Figure 2A). As the pH value increases, more dissociated PAA chains stretch and disperse in aqueous solutions, resulting in a decrease in the compact structure of PAA chains (Figure 2B). Consequently, the force between a stretched PAA chain and the tip of the AFM is so small that it is beyond the resolution limit of our technique and thus would not be detectable by AFM. As the PAA chains stretch and disperse, the pore sizes are observed to increase, albeit not conventionally, and significantly swell in PAA areas according to the AFM images with the flat surfaces of these films in different external environments. At high pH, the carboxylic acid groups were almost entirely converted to carboxylate anions, so the PAA chains were stretched considerably, and the PAA chains were all dispersed in aqueous solutions, as displayed in Figure 2C. For pH > 11.0, we were not able to obtain reliable data because of imaging problems. Ludwigs *et al.* also found a similar phenomenon in the poly(styrene)–*block*–poly(2–vinylpyridine)–*block*–poly(methacrylic acid) triblock terpolymer [18]. This phenomenon is responsible for the dissolution of entire PS–*b*–PAA microporous films in pH > 11.0 buffer solutions.

To test whether the pH response is reversible, the pore sizes of a selected sample were monitored as the pH was sequentially varied from 2.4 to 9.2 (circles) and then returned to 2.4 (triangles). The experimental results indicated there was a reversible pH response of the PS–*b*–PAA microporous solid thin film in 0.05 M sodium phosphate buffer solutions. Therefore, the pH-responsive microporous solid thin film retained a molecular memory and reverted back to a similar level as in the initial stage.

**Figure 3:**
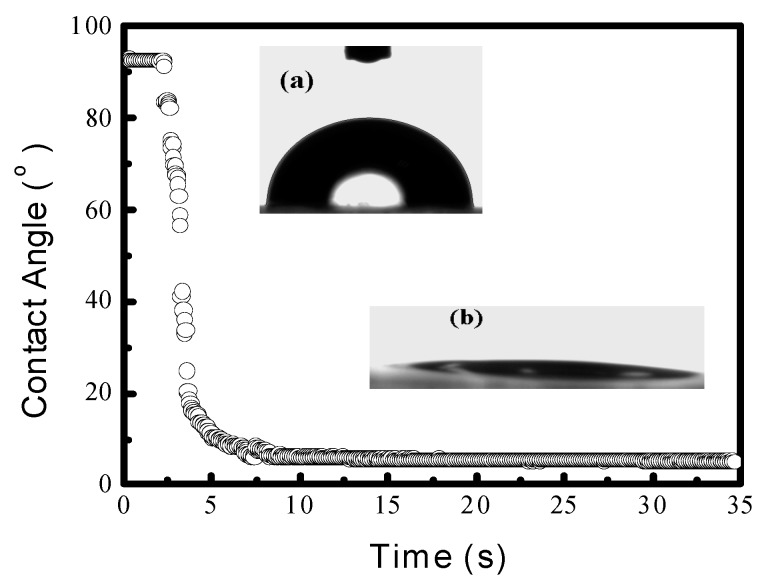
Contact angles of water on stimulus-responsive PS–*b*–PAA microporous films as a function of time. Insets are water droplet profiles on the stimulus-responsive PS–*b*–PAA microporous films in different stages: (**a**) represents a typical droplet profile in the initial stage, whereas (**b**) is a representative profile with PAA chains covering the surface stage.

Surface wetting properties rely on the surface structure of stimulus-responsive PS–*b*–PAA microporous solid thin films. Figure 3 presents the switchable wettability behavior, with an exceptionally large change in contact angle (CA), from nearly hydrophobic (CA of about 93°, as shown in Figure 3a), to super-hydrophilic (CA of near 5°, Figure 3b). The CA is consistent with that of PS [48] because PS covers the surface structure of PS–*b*–PAA microporous solid thin films in the initial stage. When the micropore film was immersed in water, the carboxylic acid groups can be partially dissociated and present more as hydrophilic carboxylate anions (–COO^−^). The hydrophilic PAA chains dramatically stretch outward and gradually cover the film surface, leading to the decoration with a continuous PAA wetting layer. The subsequent decrease of CA to a minimum value after 10 s is reasonable, given the formation of the charged brush-like hydrophilic –COO^−^ layer, which has the highest affinity for the aqueous phase. This change in conformation may be essential for the large change of CA. It seems reasonable to conclude that more hydrophilic –COO^−^ likely enhance the hydrophilic properties and hence result in the observed decrease in CA.

### 3.3. PS–b–PAA Microporous Solid Thin Film Stimulus-Responsive Dynamics

**Figure 4:**
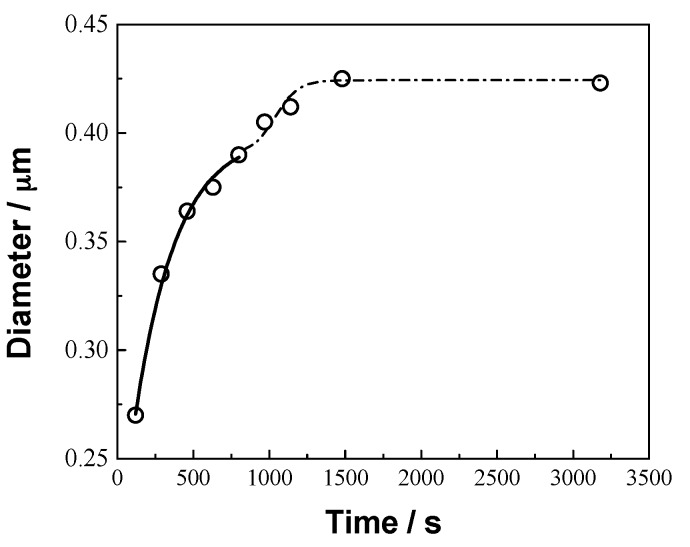
Stimulus-responsive dynamics of the pore size of the PS–*b*–PAA microporous solid thin film was observed by *in-situ* AFM in pH = 4.6 buffer solution. The solid line corresponds to a fit with Equation (1), and the dashed line is a fit with Equation (2).

To determine whether the stimulus-responsive behavior in buffer solutions is kinetic in origin, we investigated the time-responsive behavior of the selected solid thin film in pH = 4.6 buffer solution. Figure 4 shows that the pore size of the film grows quickly from 0.27 ± 0.05 to 0.42 ± 0.07 μm between 120 s and 1200 s. After 1200 s, the pore size grows much more slowly and levels off at a constant value of 0.42 ± 0.08 µm. The stimulus-responsive dynamics of the pore size is consistent with the two-stage mechanism described below.

To understand PAA stimuli-response, a two-stage model is used to describe the pH stimuli-responsive dynamics of the pore size, referred to as the pH stimuli-responsive swelling kinetics of amphiphilic block copolymers [1,47]. The dynamics is governed initially by the water rapid diffusion into the PAA micro-domains, and then by a slow relaxation of the polymer chains [18]. The initial stage dynamic stimuli-responsive behavior of the pore size is described by Equation (1) [1,47,49] and in Table 2:

(1) $$D_{t}=D_{0}+k_{1}t^{n}.$$

The most important variable in Equation (1), *n*, is the scaling exponent that describes the type of diffusion into the polymer, which is definitely not the meaning of the enlargement percentage of the pore size. Equation (1) is valid only when the enlargement fraction of the pore size is less than 0.60. For the various value *n*, *n* < 0.5 implies Fickian diffusion; 0.5 < *n* < 1.0 indicates anomalous diffusion; *n* = 1.0 describes case II transport; *n* > 1.0 indicates that the transport mechanism is super case II [1,47]. The solid line in Figure 4 is fit to the data using *n* = 1.4, suggesting that the rapid diffusion into the PAA micro-domains by adsorbing water follows super case II. For the enlargement fraction of pore size greater than 60%, the stimuli-responsive process is assumed to be dominated by molecular relaxation. The last stage sorption process is fit with Equation (2) [1,47,49]:

(2) $$\frac{D_{t}}{D_{\infty }}=1-Be^{\left(-k_{2}t\right)}.$$

The dotted line in Figure 4 is fit using *k*_2_ = 0.003 s^−1^. The two-stage model has successfully interpreted the stimuli-responsive dynamics of PS–*b*–PAA microporous solid thin film. Stimuli response will be further supported by *in-situ* AFM in the following sections.

### 3.4. The pH Effect on Electrochemical Responses of the Microporous Solid Thin Film

**Figure 5:**
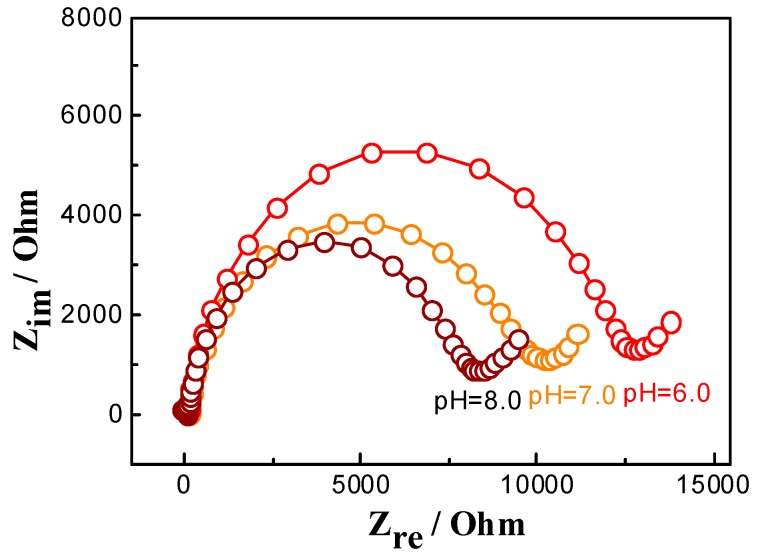
Nyquist plot (Zim *vs.* Zre) for Faradaic impedance measurements in the presence of 5.0 mM K_3_[Fe(CN)_6_]/K_4_[Fe(CN)_6_] and 0.1 M KCl with different pH values. A GCE modified by PS–*b*–PAA thin microporous film was as a work electrode, a saturated calomel electrode served was as the reference electrode and a platinum plate served was as the auxiliary electrode. The frequency range is from 10^−1^ to 10^6^ Hz with a signal amplitude of 10 mV.

Previously, we successfully fabricated microelectrode ensembles by modifying the microporous solid thin films at conventional disc electrodes [43,44] and found CTAB irreversible effects on electrochemical responses of the microporous film [50]. Electrochemical impedance spectroscopy is an important analytical tool for investigating the interface properties of modified electrode surface, which includes a semicircle and a straight line. The pore size of the semicircle at higher frequencies is corresponding to electron transfer resistance (Rct). Figure 5 shows electrochemical impedance responses of the GCE modified by the microporous thin film in hexacyanoferrate (II)/(III) solutions with different pH values. This experimental result indicated that Rct dropped dramatically with the increase of pH value in the solution, implying that the electron-transfer enhabced and micropores were enlarged in the high pH-value solution so as to enlarge the electrode area. This further evidences the change of the pore sizes of the thin microporous solid film after interactions with different acidic or basic aqueous solutions.

## 4. Conclusions

We fabricated stimulus-responsive microporous films by PS–*b*–PAA molecular self-assembly. These solid thin films exhibit different surface morphologies in response to external environmental stimuli, here involving different pH values. The reversible evolution of the microstructure in aqueous solutions over a pH range of 2.4–9.2 was investigated by *in-situ* AFM. These findings demonstrate that PAA blocks play a significant role in the amplification of stimulus-responsive behavior, and this concept is expected also to be valid for other types of weak polyelectrolytes. These observations have been explained by positing that there is no conventional PAA swelling, but the PAA chains in the micropores stretch and contract with changes in the pH of the solution environment. The successful application of the AFM technology enabling the explication of the *in-situ* tunable “switch-on/switch off” surface properties of the PS–*b*–PAA microporous solid thin film polymer in aqueous solutions of varying pH now affords us the exciting prospect of applying this microporous solid thin film to new technologies such as membrane separation, optical filters, photoelectric equipment, micro-reactors, micro-sensors, and cell culture substrates.

## Figures and Tables

**Table 1:** Equilibrium time of the pore size at different pH values.

**pH Value of the Buffer Solution**	2.4	3.5	4.6	5.1	6.8	9.2
**Time for the Formation of the Pore in Stable Size (s)**	1125	1150	1200	1550	1750	1980

**Table 2:** The meaning of the variables in Equations (1) and (2).

Name	Meaning of Variable
*D_t_*	pore size at time *t*
*D*_0_	pore size in the dry state before react with water
*D_∞_*	pore size at infinitely-great time
*k*_1_	a characteristic constant that depends on the local environment of the penetrant
*t*	the reaction time
*n*	scaling exponent that describes the type of diffusion into the polymer
*B*	a scaling constant
*k*_2_	the relaxation constant
